# Aberrant interhemispheric functional connectivity in major depressive disorder with and without anhedonia

**DOI:** 10.1186/s12888-022-04343-x

**Published:** 2022-11-08

**Authors:** Shaojia Lu, Jiamin Shao, Qian Feng, Congchong Wu, Zhe Fang, Lili Jia, Zheng Wang, Shaohua Hu, Yi Xu, Manli Huang

**Affiliations:** 1 Department of Psychiatry, The First Affiliated Hospital, Zhejiang University School of Medicine, Key Laboratory of Mental Disorder’s Management of Zhejiang Province, Zhejiang Engineering Center for Mathematical Mental Health, Hangzhou, Zhejiang China; 2 Faculty of Clinical Medicine, Zhejiang University School of Medicine, Hangzhou, Zhejiang China; 3 Department of Clinical Psychology, The Fifth Peoples’ Hospital of Lin’an District, Hangzhou, Zhejiang China

**Keywords:** Major depressive disorder, Anhedonia, Functional magnetic resonance imaging, Voxel-mirrored homotopic connectivity

## Abstract

**Objective:**

Anhedonia is a core feature of major depressive disorder (MDD), and as a subtype of depression, MDD with anhedonia may have exceptional neurobiological mechanisms. However, the neuropathology of anhedonia in MDD remains unclear. Thus, this study aimed to investigate the brain functional differences between MDD with and without anhedonia.

**Methods:**

A total of 62 individuals including 22 MDD patients with anhedonia, 20 MDD patients without anhedonia, and 20 healthy controls (HCs) were recruited for this study. All participants underwent 3.0-T functional magnetic resonance imaging scan. Voxel-mirrored homotopic connectivity (VMHC) was employed to quantitatively describe bilateral functional connectivity. Analyses of variance (ANOVA) were performed to obtain brain regions with significant differences among three groups and then post hoc tests were calculated for inter-group comparisons.

**Results:**

The ANOVA revealed significant VMHC differences among three groups in the bilateral middle temporal gyrus (MTG), superior frontal gyrus (SFG), and inferior parietal lobule (IPL) (*F* = 10.47 ~ 15.09, *p* < 0.05, *AlphaSim corrected*). Relative to HCs, MDD with anhedonia showed significantly decreased VMHC in the bilateral MTG (*t* = -5.368, *p* < 0.05, *AlphaSim corrected*), as well as increased VMHC in the bilateral SFG (*t* = -4.696, *p* < 0.05, *AlphaSim corrected*). Compared to MDD without anhedonia, MDD with anhedonia showed significantly decreased VMHC in the bilateral MTG and IPL (*t* = -5.629 ~ -4.330, *p* < 0.05, *AlphaSim corrected*), while increased VMHC in the bilateral SFG (*t* = 3.926, *p* < 0.05, *AlphaSim corrected*). However, no significant difference was found between MDD without anhedonia and HCs.

**Conclusion:**

The present findings suggest that MDD with and without anhedonia exhibit different patterns of interhemispheric connectivity. Anhedonia in MDD is related to aberrant interhemispheric connectivity within brain regions involved in the frontal–temporal-parietal circuit.

## Introduction

Major depressive disorder (MDD) is a highly heterogeneous and etiologically complex psychiatric disorder, involving a broad spectrum of psychopathology from distinct pathophysiological mechanisms [1]. Different subtypes of depression are being distinguished in the literature, among which MDD with anhedonia has drawn more and more attention in recent years [2]. Anhedonia, which used to define as a “loss of pleasure”, now reflects a broader conceptualization that includes interest and consummatory pleasure according to the *Diagnostic and Statistical Manual of Mental Disorders, fifth edition* (*DSM-5*) [3]. It has been reported that MDD with anhedonia is associated with greater severity of depressive episodes, more likelihood of psychotic symptoms, and increased suicidality [4, 5]. Moreover, it has been revealed that anhedonia in MDD predicts poorer response to pharmacological treatments for depression and poorer long-term outcomes [6, 7]. Despite the importance of anhedonia for the diagnosis and prognosis of MDD, the neural mechanisms associated with anhedonia in MDD are poorly understood.

Over a few decades, it has been suggested that clinically relevant differences in the underlying pathophysiology in patients with MDD exist [8]. In particular, as a core feature of MDD, the identification of anhedonia endophenotype for MDD may improve our understanding of the disease. Consequently, previous studies have revealed that distinct alterations of the hypothalamic–pituitary–adrenal (HPA) axis activity [9], inflammatory characteristics [10], gut microbiota abundance [11], and metabolism of brain-derived neurotrophic factor (BDNF) [12] may play a critical role for MDD patients with and without anhedonia. Taken together, these findings suggest that anhedonia in MDD may have exceptional neurobiological mechanisms and highlight the importance of studies that focus on the effect of anhedonia on biological changes in MDD.

In recent years, noninvasive neuroimaging technique like functional magnetic resonance imaging (fMRI) has been utilized to discover neurobiological underpinnings of psychiatric disorders [13]. Simultaneously, significant efforts have been made to understand the neuroimaging characteristics of anhedonia in MDD. It has been revealed that aberrant striatal and ventral striatal activity and frontostriatal connectivity abnormalities are most frequently associated with the subjective report of anhedonia [2, 14]. Nevertheless, most of these studies investigated the neural basis of anhedonia using correlation analyses of neurocircuitry with self-reported anhedonia scores [15, 16]. In addition, the majority of previous neuroimaging findings concerning anhedonia were provided by task-based fMRI studies probing reward processing [17]. It is acknowledged that task-related alterations in neural activation may just represent a small fraction (perhaps less than 5%) of the brain’s total activity, while the majority of its resources have been used on task-independent, spontaneous neural activity, and resting state fMRI (rs-fMRI) study is the best approach to evaluate it [18]. Therefore, studies examining brain functional alterations during rest in MDD patients that carefully stratify groups by anhedonia will be a complement to current findings.

In this context, the aim of the present study was to investigate brain functional changes in patients with MDD that stratify groups by anhedonia using rs-fMRI. In the present study, a novel measure, voxel-mirrored homotopic connectivity (VMHC), also named homotopic resting-state functional connectivity (FC) was applied to process the rs-fMRI data [19]. VMHC measures the synchronicity of spontaneous functional activity between geometrically consistent mirrored regions of both hemispheres [20] and focuses more on the ability to incorporate information exchange and functional interactions between the two cerebral hemispheres [21]. Consequently, VMHC has been introduced to evaluate altered interhemispheric coordination in the study of a variety of psychiatric disorders, exploring the underlying pathophysiology [22, 23]. Based on previous evidence, we hypothesized that MDD with anhedonia would exhibit unique VMHC changes in certain brain regions and the altered regions might be distinct areas that could be applied to separate anhedonic MDD from non-anhedonic MDD.

## Method

### Participants

A total of 42 patients with MDD, including 22 MDD patients with anhedonia and 20 MDD patients without anhedonia, were recruited from Department of Psychiatry, The First Affiliated Hospital, Zhejiang University School of Medicine. For assignment to the MDD with anhedonia group, MDD patients must have been experiencing anhedonia according to the Item 2 (loss of interest or pleasure) of the symptom criteria (A) for MDD in the *Diagnostic and Statistical Manual of Mental Disorders, IV Edition* (DSM-IV) and the threshold of transformed score of Snaith-Hamilton Pleasure Scale (SHAPS) [24]. Each MDD patient met the DSM-IV diagnostic criteria for current unipolar MDD episode. The inclusion criteria for MDD patients also included: 1) aged 18–45 years; 2) drug-naïve patients with first-episode depression or recurrent depression with continued withdrawal of more than 3 months; 3) total score of the 17-item Hamilton Depression Scale (HAMD-17) [25] ≥ 17; 4) right-handedness; 5) could follow the instructions to keep still during MRI scanning. Furthermore, twenty sex- and age-matched healthy controls (HCs) were recruited from local residents, hospital staffs, and students. The following exclusion criteria were applied to all subjects: 1) with a history of central nervous system diseases, such as epilepsy, serious head trauma, stroke or severe mental retardation, medical conditions, or concomitant medications likely to affect the central nervous system; 2) alcohol or drug dependence or abuse; 3) female with pregnancy; 4) with a history of psychotherapy and physical therapy, such as transcranial direct current stimulation (tDCS), transcranial magnetic stimulation (TMS), and electroconvulsive therapy (ECT); 5) contraindications to MRI scan, including retractors or braces, metallic implants, and claustrophobia. Written informed consent was obtained and this study was approved by the local Ethic Committee of The First Affiliated Hospital, Zhejiang University School of Medicine.

### Clinical assessment

**Fig. 1:**
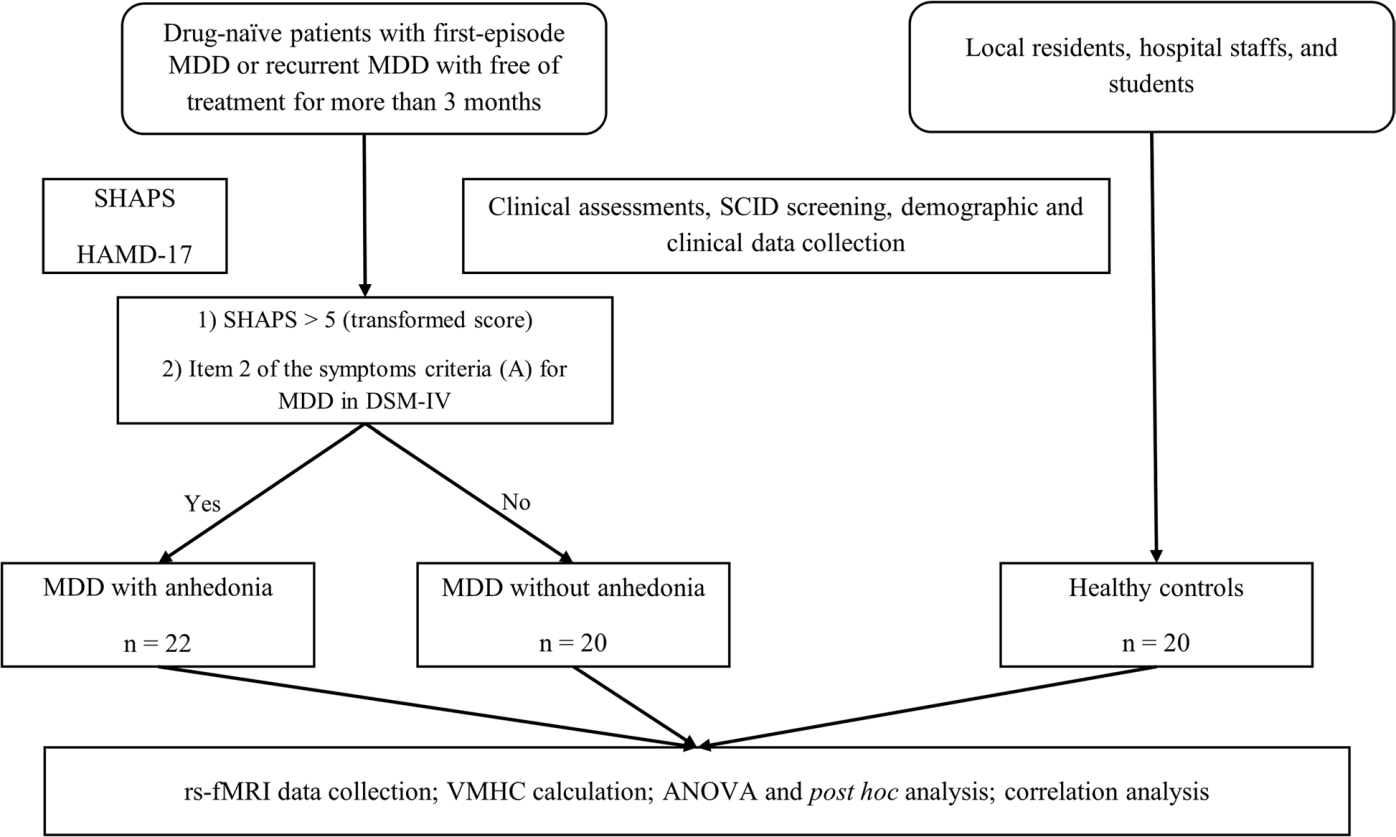
Flow chart depicting the procedure performed in this study

The demographic and clinical data was collected by a self-designed questionnaire from all the participants. The Structured Clinical Interview for DSM-IV (SCID) was used for the diagnostic assessment of MDD and further psychiatric disorders, which was also administered to each subject. The severity of depression was evaluated by using HAMD-17 [25]. Anhedonia was assessed by SHAPS [26]. This 14-item, reliable, and valid self-reporting questionnaire can yield four domains of hedonic experience, including interests and pastimes, social interactions, sensory experiences, and diet. Four answers to each item are possible: Strongly disagree, Disagree, Agree, or Strongly agree. When the participant responses ‘‘Strongly agree’’ or ‘‘Agree’’, the item is recorded a score of 0. In contrast, if the answer is ‘‘Disagree’’ or ‘‘Strongly disagree’’, the score is 1. The total score of SHAPS represents the severity of anhedonia and the sum of transformed score > 5 indicates the presence of severe anhedonia [12]. The Chinese version of SHAPS was applied in our study, which had been demonstrated to be a useful and promising instrument in assessing anhedonia for clinical patients and non-clinical individuals in the Chinese settings [24]. The procedure performed in this study was shown in Fig. 1.

### MRI acquisition

Imaging data were collected on a 3.0-T MRI scanner (Signa, HDxt, GE healthcare, USA), which is equipped with a standard birdcage head coil at the Magnetic Resonance Center of The First Affiliated Hospital, Zhejiang University School of Medicine. All subjects were asked to lie down with eyes closed and not to fall asleep. A brain volume (BRAVO) sequence was used for Sagittal 3D T1-weighted images with the following parameters: repetition time (TR) = 7.3 ms, echo time (TE) = 3.0 ms, flip angle = 7°, field of view (FOV) = 256 × 256 mm^2^, Matrix = 256 × 256, slice thickness = 1 mm, slices = 192. A gradient echo (GRE) and echo planar imaging (EPI) sequence was used to obtain rs-fMRI data with the following parameters: axial slice, TR = 2000 ms, TE = 30 ms, flip angle = 90°, FOV = 220 $\times$ 220 mm^2^, Matrix = 64 $\times$ 64, slice thickness = 4.0 mm, spacing = 0.6 mm, slice order = interleaved and bottom-up, slices = 33, measurements = 180, scan time = 6.00 min.

### Data processing and VMHC analysis

All the rs-fMRI data were preprocessed by using Data Processing Assistant for Resting-State fMRI Advanced Edition V2.2 (DPARSFA, www.restfmri.net/forum/ DPARSF) [27] toolkit with Statistical Parametric Mapping (SPM8, http://wwwfil.ion.ucl.ac.uk/spm) in MATLAB (R2010b) (MathWork, Natick, MA, USA). The steps were as follows: 1) the first 10 images were excluded to ensure steady-state longitudinal magnetization was obtained; 2) slice timing was performed; 3) realignment for head motion correction was performed and any subjects with head motion > 3.0 mm translation or > 3.0° rotation in any direction were excluded; 4) the T1-weighted images were segmented into grey matter, white matter, and cerebrospinal fluid, and then the fMRI data were normalized to the standard Montreal Neurological Institute (MNI) space; 5) the normalized images were resampled to a voxel size of 3 $\times$ 3 $\times$ 3 mm^3^; 6) the generated images were smoothed with a Gaussian kernel having a full-width at half-maximum (FWHM) value of 4 mm; and 7) linear trend removal and temporal bandpass (0.01–0.1 Hz) filtering were finally conducted. For VMHC analysis, Pearson’s correlations were computed between each time series and that of mirrored interhemispheric voxels. The correlation values were then transformed by Fisher’s z to improve their normal distribution. The obtained VMHC maps were used for statistical analyses. Further details about data analysis can be found in previous studies [21, 28], such as the VMHC computation and generation of the standard symmetric brain template. The mean framewise displacement (FD) that indicated the temporal derivative of the motion parameters was calculated by averaging FD*i* from every time point for each subject [29]. In the present samples, the mean FD ranged from 0.03 to 0.16 mm.

### Statistical analyses

The demographic and clinical data were analysed by using Statistical Package for the Social Sciences (SPSS) version 16.0 (SPSS Inc., Chicago, IL, USA). For categorical and continuous variables, the Chi-square tests (*χ*^*2*^) and analyses of variance (ANOVAs) were used for statistical analyses respectively. The level of two-tailed statistical significance was set at *p* < 0.05 for all tests.

For VMHC comparisons, one-way ANOVA was calculated on the individual imaging maps in a voxel-by-voxel manner, and according to the results of ANOVA, a mask was built. Then, post hoc analyses, which based on the mask, were performed to identify the inter-group differences. Although age, sex, and educational level did not significantly differ among three groups, they were introduced as covariates in the measures. The results were corrected for multiple comparisons to a significant level of *p* < 0.05 by DPABI AlphaSim program with 10,000 iterations combining individual voxel threshold *p* < 0.001 with a minimum cluster size > 5 voxels for VMHC. Finally, the mean VMHC values of brain regions with significant group differences were extracted by using region of interest (ROI) analyses. The association analyses between VMHC values and clinical features in MDD patients were further performed.

## Result

### Demographic and clinical characteristics

Demographic and clinical features of MDD patients and HCs in the sample are summarized in Table 1. No significant difference was found in age, sex, and years of education among three groups of subjects (all *p* > 0.05). The two groups of MDD patients did not differ with respect to illness duration and scores of HAMD-17 (all *p* > 0.05). Specifically, MDD patients with anhedonia showed higher scores of SHAPS compared with MDD patients without anhedonia (*p* < 0.05).

**Table 1:** Demographic and clinical characteristics for all subjects (*n* = 62)

	MDD with anhedonia,*n* = 22 means(SD)	MDD without anhedonia,*n* = 20 means(SD)	HCs, *n* = 20 means(SD)	Analysis *F/χ*^*2*^	*p*-values
Age (years)	28.6(7.73)	29.1(7.70)	27.7(5.07)	0.223	0.801
Gender (Male/Female)	5/17	10/10	10/10	4.387	0.112
Education years	14.4(2.02)	14.4(3.05)	15.3(1.21)	0.985	0.379
Illness duration (months)	23.4(27.5)	19.9(22.9)		0.194	0.662
Mean FD (mm)	0.06(0.03)	0.07(0.03)	0.07(0.03)	0.417	0.661
SHAPS score	37.7(7.09)	26.7(3.76)		38.14	0.000
HAMD score	25.2(3.89)	23.7(2.79)		2.097	0.155

*HAMD* Hamilton Depression Scale, *HCs* Healthy Controls *MDD* Major Depressive Disorder, *FD* framewise displacement, *SD* Standard Deviation, *SHAPS* Snaith-Hamilton Pleasure Scale

### VMHC differences among groups

**Fig. 2:**
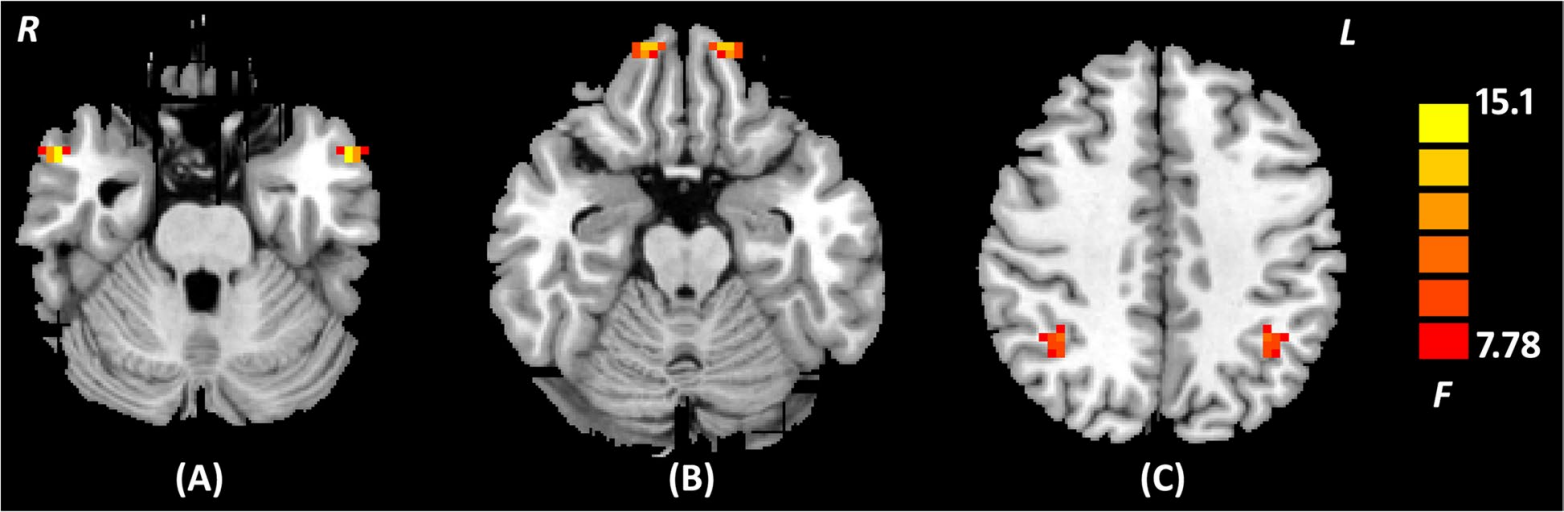
Brain regions showing significant VMHC differences among MDD with and without anhedonia and HCs (*p* < 0.05, *AlphaSim corrected*). The color scale represents *F* values. **A** Bilateral MTG; **B** Bilateral SFG; **C** Bilateral IPL

**Fig. 3:**
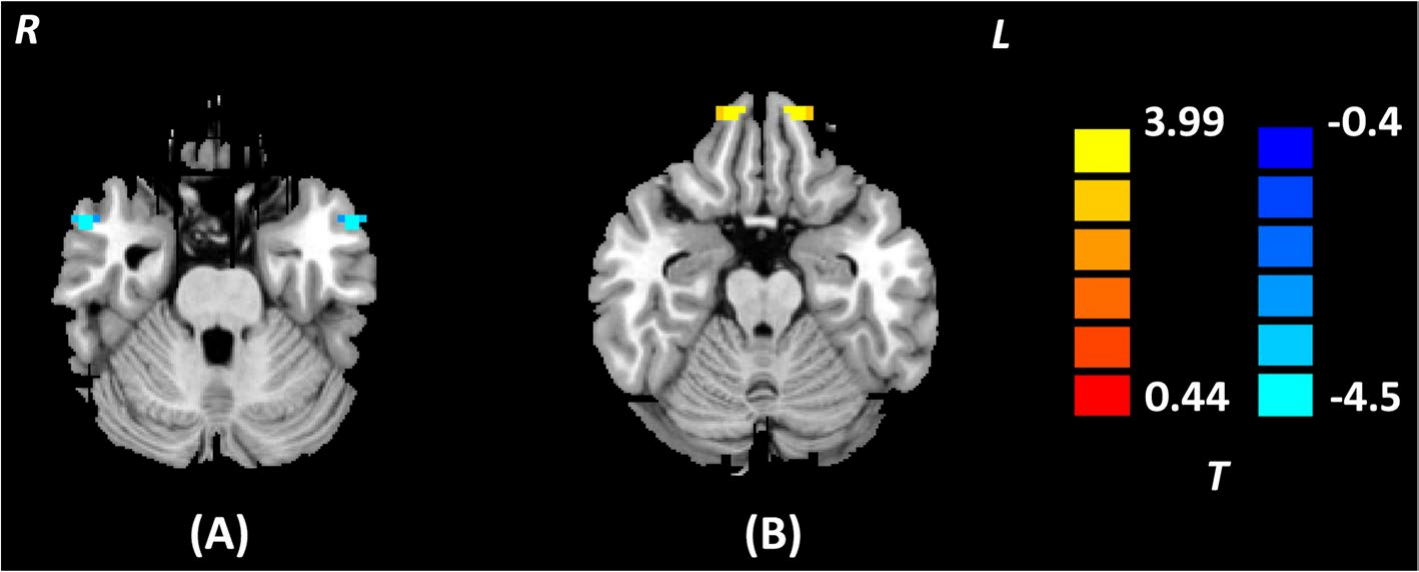
Brain regions showing significant VMHC differences between MDD with anhedonia and HCs (*p* < 0.05, *AlphaSim corrected*). The color scale represents *t* values. **A** Bilateral MTG; **B** Bilateral SFG

**Fig. 4:**
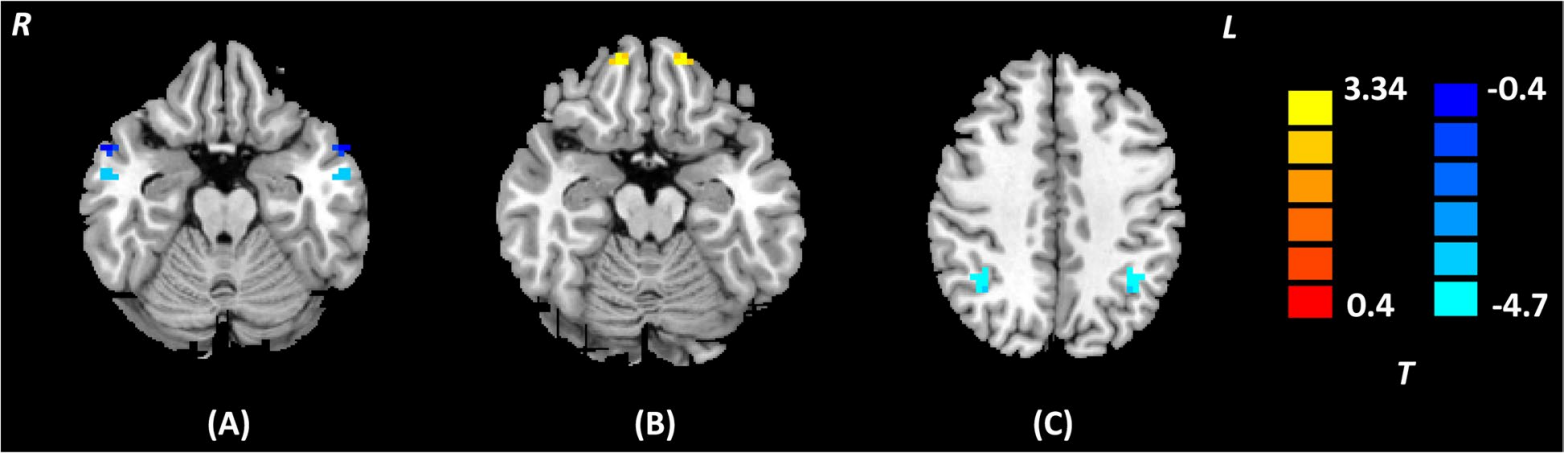
Brain regions showing significant VMHC differences between MDD with and without anhedonia (*p* < 0.05, *AlphaSim corrected*). The color scale represents *t* values. **A** Bilateral MTG; **B** Bilateral SFG; **C** Bilateral IPL

The ANOVA revealed significant VMHC differences among three groups in the bilateral middle temporal gyrus (MTG), superior frontal gyrus (SFG), and inferior parietal lobule (IPL) (Table 2 and Fig. 2). Relative to HCs, MDD with anhedonia showed significantly decreased VMHC in the bilateral MTG, as well as increased VMHC in the bilateral SFG (Table 3 and Fig. 3). Compared to MDD without anhedonia, MDD with anhedonia showed significantly decreased VMHC in the bilateral MTG and IPL, while increased VMHC in the bilateral SFG (Table 4 and Fig. 4). However, no significant difference was found between MDD without anhedonia and HCs. No significant associations were found between VMHC values of brain areas with group differences and clinical features in patients with MDD.

**Table 2:** Brain regions showing significant VMHC differences among MDD with and without anhedonia and HCs (*p* < 0.05, *AlphaSim corrected*)

Brain region	Hemisphere	Cluster size	*F* value	MNI coordinate		
				***x***	***y***	***z***
MTG	L	17	15.09	-54	9	-27
	R			54	9	27
SFG	L	20	13.84	-12	57	-21
	R			12	57	-21
IPL	L	10	10.47	-36	-51	42
	R			36	-51	42

*HCs* Healthy Controls, *IPL* Inferior Parietal Lobule, *MTG* Middle Temporal Gyrus, *MDD* Major Depressive Disorder, *MNI* Montreal Neurological Institute, *SFG* Superior Frontal Gyrus

**Table 3:** Brain regions showing significant VMHC differences between MDD with anhedonia and HCs (*p* < 0.05, *AlphaSim corrected*)

Brain region	Hemisphere	Cluster size	*t* value	MNI coordinate		
				***x***	***y***	***z***
MTG	L	17	-5.368	-54	6	-27
	R			54	6	-27
SFG	L	18	4.696	-12	57	-21
	R			12	57	-21

*HCs* Healthy Controls, *MTG* Middle Temporal Gyrus, *MDD* Major Depressive Disorder, *MNI* Montreal Neurological Institute, *SFG* Superior Frontal Gyrus

**Table 4:** Brain regions showing significant VMHC differences between MDD with and without anhedonia (*p* < 0.05, *AlphaSim corrected*)

Brain region	Hemisphere	Cluster size	*t* value	MNI coordinate		
				***x***	***y***	***z***
MTG	L	8	-4.330	-57	-6	-21
	R			57	-6	-21
SFG	L	7	3.926	-15	54	-18
	R			15	54	-18
IPL	L	10	-5.629	-36	-48	42
	R			36	-48	42

*HCs* Healthy Controls, *IPL* Inferior Parietal Lobule, *MTG* Middle Temporal Gyrus, *MDD* Major Depressive Disorder, *MNI* Montreal Neurological Institute, *SFG* Superior Frontal Gyrus

## Discussion

In this study, the whole-brain interhemispheric connectivity as measured by VHMC in MDD patients with respect to anhedonia was examined. Our results revealed that MDD patients without anhedonia was less likely to be associated with aberrant interhemispheric connectivity, but current MDD diagnosis and anhedonia combined seemed to be good predicts of altered VMHC in the SFG, MTG, and IPL. Our present findings demonstrated that MDD patients with and without anhedonia exhibited different patterns of intrinsic brain features and together with prior evidence [30], the current findings might also suggest that MDD with anhedonia could be treated as a functional subtype of depression with unique neuroimaging underpinnings. MDD is a heterogeneous disorder varying considerably in clinical manifestation, progression, treatment response and neurobiology [31]. The huge heterogeneity of MDD, especially in biological terms, presents a major obstacle for the development of new drugs and precision medicine [30]. Our findings provide insight into MDD heterogeneity in biological and clinical aspects and may represent a promising step toward the development of personalized management for MDD.

Importantly, the present study observed increased VMHC values in the SFG in MDD patients with anhedonia. Functional deficits in many regions of the prefrontal cortex (PFC) have been implicated in anhedonia [32]. It is well acknowledged that the SFG locates at the superior part of the PFC and is generally considered as a key brain area in emotion regulation-related processes [33]. Previous studies have already observed a relationship between the SFG dysfunction and anhedonia development. It has been found that disrupted cerebral functional nodal characteristics of the SFG, measured by efficiency and degree, are associated the severity of anhedonia in patients with MDD [34]. Consummatory anhedonia was found to be correlated with abnormal FC of the SFG to subregions of the anterior cingulate cortex (ACC) in a group of MDD patients [35]. Interestingly, increased SFG connectivity with the left ventral caudate over time was associated with anhedonia improvement in a prior report [36]. Furthermore, task-related fMRI studies also reported that abnormal activation across frontal regions during reward processing tasks correlated with anhedonia in MDD [37]. Together with our current discovering, all these findings may be emphasizing the crucial role of the SFG in regulating the experience of pleasure.

In the present study, MDD patients with anhedonia exhibited decreased VMHC values in the MTG. The temporal cortex has been illustrated to be involved in emotion regulation, attention control, memory processing, and social cognition [38–42], in which the MTG participated in cued attention [43]. A previous study reported that melancholic MDD showed decreased network homogeneity (NH) in the right MTG relative to HCs [44], thus, dysfunction in the MTG might be associated with anhedonia since one of the distinctive clinical presentations of melancholic MDD was anhedonia [45]. In a recent study, increased anhedonia severity was found to be correlated with the magnitude of ventral caudate FC with the cuneus and the MTG in patients with first-episode drug-naïve MDD, suggesting that the temporal area participated in reward-related behavior as well [46]. Meanwhile, the MTG is involved in the default mode network (DMN), and abnormal functional alterations of the DMN might be characteristic of anhedonia [47]. A study investigating the neural correlates of posttraumatic anhedonia symptoms demonstrated that decreased FC between the brain DMN regions specifically correlated to anhedonia symptoms [48]. In addition, impaired reward responsivity, a representation of anhedonia symptom, was associated with DMN hyperconnectivity [49] and reductions in FC within the DMN co-occurred with anhedonia improvement across two psychosocial treatments for anhedonia [50]. In view of the above-mentioned facts, we speculated that aberrant interhemispheric FC of the MTG was critical to the neurobiology of anhedonia.

Decreased VMHC values in the IPL was also found in MDD patients with anhedonia when comparing to MDD without anhedonia. The IPL, a part of the frontoparietal network, has a key role in directly representing associations between tasks and rewards, alongside directly linking cognitive control and motivational functions in the brain [51]. In a prior case report, a 71-year-old right-handed man developed an infarction in the right IPL which was associated with musical anhedonia subsequently, namely selective impairment of emotional experience in listening to music [52], suggesting the potential role of the IPL in anhedonia. In the meantime, a positive correlation was indicated between levels of social anhedonia and the thickness of the inferior parietal gyri in a non-clinical sample, which contributed to the understanding of the importance of IPL in anhedonia development [53]. Although not correlated with anhedonia severity, a very large bilateral, but right-dominant, fronto-temporo-parietal network was activated during an unexpected reward gain task in a group of patients with psychiatric symptoms, exhibiting a full range of anhedonia [54]. Consistently, our novel results added to the literature on involvement of the frontal–temporal-parietal circuit dysfunction in MDD patients with anhedonia.

Certain limitations of our study should be mentioned. First, our sample size was relatively small, thus, the current findings should be understood as preliminary and tentative, which required to be verified in larger cohorts of MDD patients with anhedonia. Second, previous studies have found that different types of anhedonia may have specifical impacts on neural function [14], however, due to the modest sample size, we did not investigate the effects of different types of anhedonia on brain functional alterations. Third, this study was a cross-sectional design, the causal relationship of altered brain function with anhedonia in MDD could not be directly determined.

## Conclusion

In conclusion, the present findings suggest that MDD with and without anhedonia exhibit different patterns of interhemispheric connectivity. Anhedonia in MDD is related to aberrant interhemispheric connectivity within brain regions involved in the frontal–temporal-parietal circuit.

## Data Availability

The datasets generated and/or analyzed during the current study are not publicly available due to privacy and ethical restrictions but are available from the corresponding author on reasonable request.

## References

[1] Tian H, Hu Z, Xu J, Wang C (2022). The molecular pathophysiology of depression and the new therapeutics. MedComm.

[2] Pizzagalli DA (2022). Toward a better understanding of the mechanisms and pathophysiology of Anhedonia: are we ready for translation?. Am J Psychiatry.

[3] Rizvi SJ, Pizzagalli DA, Sproule BA, Kennedy SH (2016). Assessing anhedonia in depression: Potentials and pitfalls. Neurosci Biobehav Rev.

[4] Halbreich U, Kahn LS (2007). Atypical depression, somatic depression and anxious depression in women: are they gender-preferred phenotypes?. J Affect Disord.

[5] Gabbay V, Johnson AR, Alonso CM, Evans LK, Babb JS, Klein RG (2015). Anhedonia, but not irritability, is associated with illness severity outcomes in adolescent major depression. J Child Adolesc Psychopharmacol.

[6] McMakin DL, Olino TM, Porta G, Dietz LJ, Emslie G, Clarke G, Wagner KD, Asarnow JR, Ryan ND, Birmaher B (2012). Anhedonia predicts poorer recovery among youth with selective serotonin reuptake inhibitor treatment-resistant depression. J Am Acad Child Adolesc Psychiatry.

[7] Winer ES, Nadorff MR, Ellis TE, Allen JG, Herrera S, Salem T (2014). Anhedonia predicts suicidal ideation in a large psychiatric inpatient sample. Psychiatry Res.

[8] Antonijevic IA (2006). Depressive disorders – is it time to endorse different pathophysiologies?. Psychoneuroendocrinology.

[9] Jacobson L (2014). Hypothalamic-pituitary-adrenocortical axis: neuropsychiatric aspects. Compr Physiol.

[10] Tang W, Liu H, Chen L, Zhao K, Zhang Y, Zheng K, Zhu C, Zheng T, Liu J, Wang D (2021). Inflammatory cytokines, complement factor H and anhedonia in drug-naive major depressive disorder. Brain Behav Immun.

[11] Mason BL, Li Q, Minhajuddin A, Czysz AH, Coughlin LA, Hussain SK, Koh AY, Trivedi MH (2020). Reduced anti-inflammatory gut microbiota are associated with depression and anhedonia. J Affect Disord.

[12] Wu C, Lu J, Lu S, Huang M, Xu Y (2020). Increased ratio of mature BDNF to precursor-BDNF in patients with major depressive disorder with severe anhedonia. J Psychiatr Res.

[13] Keilholz S, Caballero-Gaudes C, Bandettini P, Deco G, Calhoun V (2017). Time-resolved resting-state functional magnetic resonance imaging analysis: current status, challenges, and new directions. Brain connectivity.

[14] Wang S, Leri F, Rizvi SJ (2021). Anhedonia as a central factor in depression: Neural mechanisms revealed from preclinical to clinical evidence. Prog Neuropsychopharmacol Biol Psychiatry.

[15] Young CB, Chen T, Nusslock R, Keller J, Schatzberg AF, Menon V (2016). Anhedonia and general distress show dissociable ventromedial prefrontal cortex connectivity in major depressive disorder. Transl Psychiatry.

[16] Hwang JW, Xin SC, Ou YM, Zhang WY, Liang YL, Chen J, Yang XQ, Chen XY, Guo TW, Yang XJ (2016). Enhanced default mode network connectivity with ventral striatum in subthreshold depression individuals. J Psychiatr Res.

[17] Schiller CE, Walsh E, Eisenlohr-Moul TA, Prim J, Dichter GS, Schiff L, Bizzell J, Slightom SL, Richardson EC, Belger A (2022). Effects of gonadal steroids on reward circuitry function and anhedonia in women with a history of postpartum depression. J Affect Disord.

[18] Fox MD, Raichle ME (2007). Spontaneous fluctuations in brain activity observed with functional magnetic resonance imaging. Nat Rev Neurosci.

[19] Kelly C, Zuo XN, Gotimer K, Cox CL, Lynch L, Brock D, Imperati D, Garavan H, Rotrosen J, Castellanos FX (2011). Reduced interhemispheric resting state functional connectivity in cocaine addiction. Biol Psychiatry.

[20] Hu G, Ge H, Yang K, Liu D, Liu Y, Jiang Z, Hu X, Xiao C, Zou Y, Liu H (2022). Altered static and dynamic voxel-mirrored homotopic connectivity in patients with frontal Glioma. Neuroscience.

[21] Zuo XN, Kelly C, Di Martino A, Mennes M, Margulies DS, Bangaru S, Grzadzinski R, Evans AC, Zang YF, Castellanos FX (2010). Growing together and growing apart: regional and sex differences in the lifespan developmental trajectories of functional homotopy. The Journal of neuroscience : the official journal of the Society for Neuroscience.

[22] Zhang Y, Mu Y, Li X, Sun C, Ma X, Li S, Li L, Zhang Z, Qi S (2022). Improved interhemispheric functional connectivity in postpartum depression disorder: associations with individual target-transcranial magnetic stimulation treatment effects. Front Psych.

[23] Yang G, Zhang S, Zhou Y, Song Y, Hu W, Peng Y, Shi H, Zhang Y (2022). Increased resting-state interhemispheric functional connectivity of striatum in first-episode drug-naive adolescent-onset schizophrenia. Asian J Psychiatr.

[24] Liu WH, Wang LZ, Zhu YH, Li MH, Chan RC (2012). Clinical utility of the Snaith-Hamilton-Pleasure scale in the Chinese settings. BMC Psychiatry.

[25] Hamilton M (1960). A rating scale for depression. J Neurol Neurosurg Psychiatry.

[26] Snaith RP, Hamilton M, Morley S, Humayan A, Hargreaves D, Trigwell P (1995). A scale for the assessment of hedonic tone the Snaith-Hamilton Pleasure Scale. The British journal of psychiatry : the journal of mental science.

[27] Chao-Gan Y, Yu-Feng Z (2010). DPARSF: a matlab toolbox for "Pipeline" data analysis of resting-State fMRI. Front Syst Neurosci.

[28] Li HJ, Xu Y, Zhang KR, Hoptman MJ, Zuo XN (2015). Homotopic connectivity in drug-naive, first-episode, early-onset schizophrenia. J Child Psychol Psychiatry.

[29] Power JD, Barnes KA, Snyder AZ, Schlaggar BL, Petersen SE (2012). Spurious but systematic correlations in functional connectivity MRI networks arise from subject motion. Neuroimage.

[30] Wang Y, Tang S, Zhang L, Bu X, Lu L, Li H, Gao Y, Hu X, Kuang W, Jia Z (2021). Data-driven clustering differentiates subtypes of major depressive disorder with distinct brain connectivity and symptom features. The British journal of psychiatry : the journal of mental science.

[31] Chahal R, Gotlib IH, Guyer AE (2020). Research Review: Brain network connectivity and the heterogeneity of depression in adolescence - a precision mental health perspective. J Child Psychol Psychiatry.

[32] Der-Avakian A, Markou A (2012). The neurobiology of anhedonia and other reward-related deficits. Trends Neurosci.

[33] Wang S, Zhao Y, Zhang L, Wang X, Cheng B, Luo K, Gong Q (2019). Stress and the brain: Perceived stress mediates the impact of the superior frontal gyrus spontaneous activity on depressive symptoms in late adolescence. Hum Brain Mapp.

[34] Zhang L, Li Z, Lu X, Liu J, Ju Y, Dong Q, Sun J, Wang M, Liu B, Long J et al: High efficiency of left superior frontal gyrus and the symptom features of major depressive disorder. Zhong nan da xue xue bao Yi xue ban = Journal of Central South University Medical sciences 2022, 47(3):289–300. 10.11817/j.issn.1672-7347.2022.210743 PMC10930058 35545321

[35] Wu Z, Fang X, Yu L, Wang D, Liu R, Teng X, Guo C, Ren J, Zhang C (2022). Abnormal functional connectivity of the anterior cingulate cortex subregions mediates the association between anhedonia and sleep quality in major depressive disorder. J Affect Disord.

[36] Yang X, Huang J, Roser ME, Xie G (2022). Anhedonia reduction correlates with increased ventral caudate connectivity with superior frontal gyrus in depression. J Psychiatr Res.

[37] Borsini A, Wallis ASJ, Zunszain P, Pariante CM, Kempton MJ (2020). Characterizing anhedonia: a systematic review of neuroimaging across the subtypes of reward processing deficits in depression. Cogn Affect Behav Neurosci.

[38] Friedman L, Kenny JT, Wise AL, Wu D, Stuve TA, Miller DA, Jesberger JA, Lewin JS (1998). Brain activation during silent word generation evaluated with functional MRI. Brain Lang.

[39] Giraud AL, Kell C, Thierfelder C, Sterzer P, Russ MO, Preibisch C, Kleinschmidt A (2004). Contributions of sensory input, auditory search and verbal comprehension to cortical activity during speech processing. Cereb Cortex.

[40] Hesling I, Clement S, Bordessoules M, Allard M (2005). Cerebral mechanisms of prosodic integration: evidence from connected speech. Neuroimage.

[41] Sato W, Toichi M, Uono S, Kochiyama T (2012). Impaired social brain network for processing dynamic facial expressions in autism spectrum disorders. BMC Neurosci.

[42] Xu J, Lyu H, Li T, Xu Z, Fu X, Jia F, Wang J, Hu Q (2019). Delineating functional segregations of the human middle temporal gyrus with resting-state functional connectivity and coactivation patterns. Hum Brain Mapp.

[43] Corbetta M, Shulman GL (2002). Control of goal-directed and stimulus-driven attention in the brain. Nat Rev Neurosci.

[44] Cui X, Guo W, Wang Y, Yang TX, Yang XH, Gong J, Tan C, Xie G (2017). Aberrant default mode network homogeneity in patients with first-episode treatment-naive melancholic depression. International journal of psychophysiology : official journal of the International Organization of Psychophysiology.

[45] Parker G, Fink M, Shorter E, Taylor MA, Akiskal H, Berrios G, Bolwig T, Brown WA, Carroll B, Healy D (2010). Issues for DSM-5: whither melancholia? The case for its classification as a distinct mood disorder. Am J Psychiatry.

[46] Yang XH, Tian K, Wang DF, Wang Y, Cheung EFC, Xie GR, Chan RCK (2017). Anhedonia correlates with abnormal functional connectivity of the superior temporal gyrus and the caudate nucleus in patients with first-episode drug-naive major depressive disorder. J Affect Disord.

[47] Yang ZY, Zhang RT, Li Y, Wang Y, Wang YM, Wang SK, Ongur D, Cheung EFC, Chan RCK (2019). Functional connectivity of the default mode network is associated with prospection in schizophrenia patients and individuals with social anhedonia. Prog Neuropsychopharmacol Biol Psychiatry.

[48] Li G, Cao C, Fang R, Liu P, Luo S, Liberzon I, Wang L (2021). Neural correlates of posttraumatic anhedonia symptoms: decreased functional connectivity between ventral pallidum and default mode network regions. J Psychiatr Res.

[49] Sharma A, Wolf DH, Ciric R, Kable JW, Moore TM, Vandekar SN, Katchmar N, Daldal A, Ruparel K, Davatzikos C (2017). Common dimensional reward deficits across mood and psychotic disorders: a connectome-wide association study. Am J Psychiatry.

[50] Cernasov P, Walsh EC, Kinard JL, Kelley L, Phillips R, Pisoni A, Eisenlohr-Moul TA, Arnold M, Lowery SC, Ammirato M (2021). Multilevel growth curve analyses of behavioral activation for anhedonia (BATA) and mindfulness-based cognitive therapy effects on anhedonia and resting-state functional connectivity: Interim results of a randomized trial(). J Affect Disord.

[51] Wisniewski D, Reverberi C, Momennejad I, Kahnt T, Haynes JD (2015). The role of the Parietal Cortex in the representation of task-reward associations. J Neurosci.

[52] Satoh M, Nakase T, Nagata K, Tomimoto H (2011). Musical anhedonia: selective loss of emotional experience in listening to music. Neurocase.

[53] Wang Y, Deng Y, Fung G, Liu WH, Wei XH, Jiang XQ, Lui SS, Cheung EF, Chan RC (2014). Distinct structural neural patterns of trait physical and social anhedonia: evidence from cortical thickness, subcortical volumes and inter-regional correlations. Psychiatry Res.

[54] Bradley KAL, Case JAC, Freed RD, Stern ER, Gabbay V (2017). Neural correlates of RDoC reward constructs in adolescents with diverse psychiatric symptoms: a reward flanker task pilot study. J Affect Disord.

